# Analysis of *STAG3* variants in Chinese non-obstructive azoospermia patients with germ cell maturation arrest

**DOI:** 10.1038/s41598-021-89559-9

**Published:** 2021-05-12

**Authors:** Wen Liu, Xuan Gao, Haobo Zhang, Ran Liu, Yongzhi Cao, Ruimei Yu, Ge Fang, Jinlong Ma, Shidou Zhao

**Affiliations:** 1 Center for Reproductive Medicine, Cheeloo College of Medicine, Shandong University, 44 Wenhua Xi Road, Jinan, 250012 Shandong China; 2 Key Laboratory of Reproductive Endocrinology of Ministry of Education, Shandong University, Jinan, 250012 Shandong China; 3 Shandong Key Laboratory of Reproductive Medicine, Jinan, 250012 Shandong China; 4 Shandong Provincial Clinical Research Center for Reproductive Health, Jinan, 250012 Shandong China; 5 National Research Center for Assisted Reproductive Technology and Reproductive Genetics, Shandong University, Jinan, 250012 Shandong China

**Keywords:** Genetics, Molecular biology, Molecular medicine

## Abstract

STAG3 is essential for male meiosis and testis of male *Stag3*^−/−^ mice shows the histopathological type of germ cell maturation arrest (MA). Whether variants of the *STAG3* gene exist in Chinese idiopathic non-obstructive azoospermia (NOA) patients needs to be determined. We recruited 58 Chinese NOA men with MA who underwent testis biopsy and 192 fertile men as the control group. The 34 exons of the *STAG3* gene were amplified using polymerase chain reaction (PCR) and sequenced. We identified eight novel single nucleotide polymorphisms (SNPs), including two missense SNPs (c.433T > C in exon2 and c.553A > G in exon3), three synonymous SNPs (c.539G > A, c.569C > T in exon3, and c.1176C > G in exon8), and three SNPs in introns. The allele and genotype frequencies of the novel and other SNPs have no significant differences between two groups. Our results indicated that variants in the coding sequence of the *STAG3* gene were uncommon in NOA patients with MA in Chinese population. Future studies in large cohorts of different ethnic populations will be needed to determine the association between the *STAG3* gene and NOA.

## Introduction

Infertility affects approximately 10–15% couples who consider having offspring^1^. Roughly, 30–40% of all cases could be attributed to male origin, 30–40% to female origin, and the remainder involves both problems^2^. The male infertility was proposed to have several etiologies, including endocrine disorders, spermatic duct obstruction, cryptorchidism, testicular damage, cytogenetic abnormalities and Y-chromosome microdeletion^3^. Azoospermia is a form of male infertility that affects 10–20% of infertile men^4^, and nearly 50% of idiopathic azoospermia cases are considered to have a genetic basis^5,6^.

There are two types of azoospermia, i.e., obstructive azoospermia and non-obstructive azoospermia (NOA)^7,8^. According to the testicular biopsy, NOA have four histopathological types, including normal spermatogenesis, hypospermatogenesis, germ cell maturation arrest (MA) and Sertoli cell only syndrome^2,9,10^. Many mouse models have linked hundreds of genes with azoospermia, but only a few studies have identified gene variants in humans with NOA, such as *SYCP3, NR5A1, TEX11,CYP1A1,TDRD9, SOHLH1, USP26, ZMYND15 and PIWIL4*^11–18^. MA is a form of azoospermia in which the cessation occurred at stage of germ cell formation and may have its own specific etiology^19,20^.

The stromal antigen 3 (STAG3) is involved in formation of cohesin core with three other proteins including SMC1β and two α-kleisins (RAD21L and REC8), and required for synaptonemal complex formation during meiosis^21^. *Stag3*^*–/–*^ male mice showed no overt phenotype apart from sterility, which is due to azoospermia and meiotic arrest^21,22^. Notably, *Stag3*^–/–^ spermatocytes only reached zygotene-like stage of prophase I, and apoptosis occurred^23^. These results suggest that *STAG3* plays an essential role in meiosis and may be a candidate gene for NOA patients with MA^24^. In this study, we investigated whether perturbations of the *STAG3* gene were present in Chinese idiopathic NOA patients with MA histopathology.

## Methods

### Participants

In this study, male patients newly diagnosed with idiopathic NOA were recruited from the Center for Reproductive Medicine, Shandong University, from January 2014 to December 2018. All NOA patients were diagnosed on the basis of an andrological examination that included medical history, ultrasound, physical examination, hormone analysis, semen analysis, karyotype testing, and Y chromosome microdeletion screening. Subjects with known reasons or any relevant history may account for their infertility, such as childhood disease, cryptorchidism, environmental exposure, radiation, heat and other negative environmental exposure, varicocele, chromosomal abnormalities, hypogonadotropic hypogonadism, obstructive azoospermia, repeated infections, iatrogenic infertility, testicular trauma, abnormal karyotype, or Y-chromosome microdeletions, epididymitis, epididymo-orchitis, orchitis and/or sexualiy transmitted infections^25–27^ were excluded.

According to the WHO recommendations and standards^28^, after two or more inspections of semen, testicular biopsies were performed in patients without available sperm. Biopsy samples were immersed in Bouin’s fluid and then sent for histopathology examination. MA histopathology in our study exhibited that spermatogenesis blocked at the spermatocyte stage (Supplemental Fig. S1). Participants include 58 Chinese MA patients, and their mean age was 28 ± 4.1 years. A total of 192 fertile men with normal sperm concentrations were used as control group, and their mean age was 29 ± 4.2 years. All samples were treated according to the National Regulation of Clinical Sampling in China. Informed consent was obtained from all participants. The study was approved by the Institutional Review Board of Reproductive Medicine of Shandong University on October 11, 2014 (document No. 42).

### Polymerase chain reaction (PCR) and sequencing analysis

Genomic DNA from 58 MA patients and 192 control samples were extracted from peripheral blood. Thirty-four exons of the *STAG3* gene (RefSeqGene NG_034114.1) were amplified by PCR using 26 pairs of primers (Table 1). PCR mix included Buffer (Mg^2+^ Plus), 2.5 mM dNTP Mixture, 5 μM of forward primer, 5 μM of reverse primer, DNA polymerase Taq (Hot Start Version), dH_2_O and genomic DNA in a final volume of 20 μl. PCR conditions were as follows: pre-denaturation 5 min at 95 °C, 35 cycles of denaturation 30 s at 95 °C, annealing 30 s at 58 °C (60 °C for exons 14, 19, and 20), and elongation 45 s at 72 °C, and finally end-elongation 7 min at 72 °C. PCR products were firstly analyzed by Agarose gel electrophoresis and then sequenced on an automated sequencer (PRISM 310; Applied Biosystems).

**Table 1:** The STAG3 gene-specific primer sequences.

Number	Primer ID	Primer sequences (5′-3′)	Primer reverse sequences (5′-3′)	Product size (bp)
1	Exon1	AAATAGGGGCGTGGTCTCC	AAGATTCCAGAAAAGCGCGG	463
2	Exon2	GAGAAGTGCTGTGGTAGGAG	GGCCACACAATGCAACATCT	433
3	Exon3	ATGGAGGGAATAGGGTGGTT	GTTCACGCCATTCTCCTGC	427
4	Exon4	ACCAAGCGTTAATGTCACTGT	TGGTATCAACAGAGGTGAGACA	437
5	Exon5	CCTCCCAGGGTTGCTACTTA	GGCTGGGAATTAGAAAGGGG	327
6	Exon6	GGTCTTCTCATTCCCCACCT	AGGATCCTGGTCATCTTCTTCC	438
7	Exon7	TGACATCCAAGCCCCTATGA	CCAAGATGCAGGTAGGAAAGA	436
8	Exon8	TCCTCTCTCCTCTGACCTCA	AAGGGAAGGAAGAAGCAGGG	433
9	Exon9	TAACCCGTTTCTCCCTGTCC	ATTCCATAACCAAAGGCCAGC	433
10	Exon10	TGAGTTTGGAGAGAGGGTGG	CGGAAAGGGAAACTGACTCG	436
11	Exon11,12	GGGCGAGTAGAGTGTGGTTA	GGAAGGGCAAAGGTCTGAGA	513
12	Exon13	TTTCTGCTTTTCTGTGGGCA	GCAGCAGATGGAGGAGAGAA	438
13	Exon14	TGTTTCCTGTTGTGCTGAGC	TCCTATGCACAACAGCCAGA	431
14	Exon15,16	TAACTCCCCATGCACGTTCT	GCTGACCTACCCACTCTACC	503
15	Exon17,18	CCCTGCACCAGTGTTTCTTT	AAGCAGCAAGGTATAGGAAATCT	427
16	Exon19,20	CCACAGCACACCATCTTCTG	ATGGGGAAGAGGAGGAAAGC	512
17	Exon21	AGTGGCTTTCCTCCTCTTCC	CCAACCCATCTCTAGCCTGT	417
18	Exon22,23	TCCCTCTCCTAACCCAAACC	GATACTCGCCTTGTTGCTCC	436
19	Exon24,25-F	CTCAAGTGGGAGCAACAAGG	GGCTCACATGGAAGGCAAAA	387
20	Exon26	CTTCCCCACTCTTTCCCCTC	CTGAGTGGCTGAGGGTAGAC	436
21	Exon27	CTGGACTTCTCTGTTTCCGC	GGACACAACCTGCAACCAAT	423
22	Exon28,29	GGAGGGAAGTGGGAAGAGAC	TACCCACACACAGCACCCTA	500
23	Exon30	CCCTGGGCTGTGGTTAATGT	ACACCCAGATTCCCTCCATG	428
24	Exon31,32	TGATCCTGCTTCATTCCCAG	CTTGAGAGAGAAGGGCAGGG	559
25	Exon33	TTTGCGAAGTGACAGGAGTG	TTTGATGAGTGCACGGGTTG	407
26	Exon34	CGTTGCTGTGTCCTGTGTAT	GACCAAGAACCTGACCTCCA	505

### Statistical analysis

The Sanger sequencing data were analyzed with Sequencer 4.9 software (Gene Codes Corporation, USA). Statistical analyses were carried out by the Statistical Package for Social Science for Windows (SPSS, version 22.0, IBM Corp., USA). The chi-squared test or Fisher's exact test was used when appropriate, and *P* < 0.05 was considered statistically significant.

### Ethics approval and guideline statement

The study was approved by the Institutional Review committee of Reproductive Medicine of Shandong University on October 11, 2014 (document No. 42). All methods were carried out in accordance with relevant guidelines and regulations.

### Consent for publication

The publication consent was obtained from all participants.

## Results

We sequenced the STAG3 gene in 58 patients with idiopathic NOA with MA histopathology and the control 192 fertile men. As shown in Table 2, we found 12 single nucleotide polymorphisms (SNPs), including 4 known SNPs and 8 novel SNPs. The 8 novel SNPs included 2 missense SNPs (c.433T > C in exon2 and c.553A > G in exon3), 3 synonymous SNPs (c.539G > A, c.569C > T in exon3, and c.1176C > G in exon8), and 3 SNPs in introns region. The allele and genotype frequencies of all SNPs have no significant differences between the cases and control group. No plausible variants were identified.

**Table 2:** Allele and genotype frequencies of SNPs in Chinese men with MA (n = 58).

Number	Location	dbSNP ID	Sequence variation	Amino acid /protein variation	Allele	Allele frequency (n)		Genotype	Genotype frequency (n)	
						MA	Control		MA	Control
1	5′UTR	rs188290003	c.283C > A	–	C	99.1% (115)	99.2% (381)	CC	98.3% (57)	98.4% (189)
					A	0.9% (1)	0.8% (3)	CA	1.7% (1)	1.6% (3)
								AA	(0)	(0)
2	Exon2	Novel	c.433T > C	Missense p.Val 9Ala	T	59.5% (69)	60.2% (231)	TT	20.7% (12)	20.3% (39)
					C	40.5% (47)	39.8% (153)	TC	77.6% (45)	79.7% (153)
								CC	1.7% (1)	0 (0)
3	Exon3	Novel	c.539G > A	Synonymous p.Leu44Leu	G	68.1% (79)	69.0% (265)	GG	37.9% (22)	38.1% (73)
					A	31.9% (37)	31.0% (119)	GA	60.3% (35)	60.9% (117)
								AA	1.7% (1)	1.0% (2)
4	Exon3	Novel	c.553A > G	Missense p.Asp49Gly	A	69.8% (81)	71.4% (274)	AA	39.7% (23)	42.7% (82)
					G	30.2% (35)	28.6% (110)	AG	60.3% (35)	57.3% (110)
								GG	0 (0)	0 (0)
5	Exon3	Novel	c.569C > T	Synonymous p.Asp54Asp	C	69.0% (80)	70.8% (272)	CC	39.7% (23)	42.2% (81)
					T	31.0% (36)	29.2% (112)	CT	58.6% (34)	57.3% (110)
								TT	1.7% (1)	0.5% (1)
6	Intron3	Novel	c.626 + 59C > T	–	C	69.8% (81)	71.4% (274)	CC	43.2% (25)	43.2% (83)
					T	30.2% (35)	28.6% (110)	CT	53.4% (31)	56.3% (108)
								TT	3.4% (2)	0.5% (1)
7	Exon8	Novel	c.1176C > G	Synonymous p.Glu256Glu	C	70.7% (82)	70.8% (272)	CC	41.4% (24)	41.7% (80)
					G	29.3% (34)	29.2% (112)	CG	58.6% (34)	58.3% (112)
								GG	0 (0)	0 (0)
8	Exon13	rs3735241	c.1772A > T	Synonymous p.Pro455Pro	A	69.0% (80)	68.2% (262)	AA	46.6% (27)	47.4% (91)
					T	31.0% (36)	31.8% (122)	AT	44.8% (26)	41.7% (80)
								TT	8.6% (5)	10.9% (21)
9	Intron15	Novel	c.1727 + 129G > A	–	G	99.1% (115)	99.2% (381)	GG	98.3% (57)	99.0% (190)
					A	0.9% (1)	0.8% (3)	GA	1.7% (1)	0.5% (1)
								AA	0 (0)	0.5% (1)
10	Exon24	rs1043915	c.2852T > A	Synonymous p.Ile815Ile	T	61.2% (71)	57.0% (219)	TT	32.8% (19)	27.6% (53)
					A	38.8% (45)	43.0% (165)	TA	56.9% (33)	58.9% (113)
								AA	10.3% (6)	13.5% (26)
11	Intron33	Novel	c.3823 + 36C > G	–	C	61.2% (71)	63.6% (244)	CC	32.8% (19)	38.1% (73)
					G	38.8% (45)	36.4% (140)	CG	56.9% (33)	51.0% (98)
								GG	10.3% (6)	10.9% (21)
12	3′UTR	rs1052482	c.4030A > T	–	A	61.2% (71)	58.6% (225)	AA	32.8% (19)	31.8% (61)
					T	38.8% (45)	41.4% (159)	AT	56.9% (33)	53.6% (103)
								TT	10.3% (6)	14.6% (28)

*SNP* single nucleotide polymorphism, *MA* germ cell maturation arrest, – not applicable.

## Discussion

The development of male gametogenesis includes the differentiation of spermatogonia, the process of spermatocyte meiosis, and spermatogenesis^29,30^. Meiosis is a critical stage in gametogenesis, in which alignment and synapsis of chromosome pairs occur, allowing the recombination of the maternal and paternal genomes^31^. Many of the gene variants in this process could have profound effects on gametogenesis and lead to male infertility^22^. Many gene knockout mouse models showed meiotic arrest in infertility, suggesting that they are candidate genes for NOA with MA histopathology^32^.

The *STAG3* gene encodes a critical subunit of the meiosis-specific cohesin complex, ensures sister chromatid cohesion and enables correct synapsis and segregation of homologous chromosomes during meiosis^19,27^. While variant in *STAG3* was identified in premature ovarian failure and oocytes in *Stag3*^*–/–*^ female mice were arrested at early prophase I, the knockout male mice were also infertile and showed meiotic arrest and azoospermia^33,34^. These findings indicated that *STAG3* may be a potential candidate gene for NOA in human. In this study, we analyzed the *STAG3* gene in 58 Chinese NOA patients with MA histopathology, which is coincided with the phenotype of the gene knockout mice^35^. Eight novel SNPs were identified, including two missense SNPs, three synonymous SNPs and three SNPs in intron region. Our findings suggest that variants in coding region of the *STAG3* gene are uncommon in NOA patients with MA histopathology in China. However, it has been reported that two SNPs (rs1727130 and rs1052482) located in the 3’-UTR of the *STAG3* gene were identified to be associated with NOA in Korean population^36^. Furthermore, homozygous or compound-heterozygous variants of the *STAG3* gene have been identified in NOA patients from Germany, Spain, and Australia^37–39^. In this study, we did not identify the same variants which may be due to the small sample size and ethnic diversity. Consistently, whole-exome sequencing was performed in 314 Han Chinese patients with unrelated NOA and Severe Oligozoospermia, but no deleterious variants were found in *STAG3*^40^.

## Conclusions

The present study investigated variants in *STAG3* in a cohort of idiopathic NOA with MA histopathology, and found no pathogenic variants. Our results suggest that variants in the *STAG3* gene may not be responsible for NOA with MA in Chinese population. However, due to ethnic diversity, the exact role of STAG3 in the pathogenesis of NOA needs to be explored in large samples and other populations in the future.

## Supplementary Information

Supplementary Information.

## Data Availability

The datasets used and analyzed during the current study are available from the corresponding author on reasonable request.

## Abbreviations

MA: Germ cell maturation arrest
NOA: Non-obstructive azoospermia
PCR: Polymerase chain reaction
SNPs: Single nucleotide polymorphisms
STAG3: Stromal antigen 3
SPSS: Statistical Package for Social Science for Windows
