# Supplementary figures and images for: Analysis of STAG3 variants in Chinese non-obstructive azoospermia patients with germ cell maturation arrest
Source: Sci Rep. 2021 May 12;11:10077. doi: 10.1038/s41598-021-89559-9 (PMC8115624; doi:10.1038/s41598-021-89559-9)

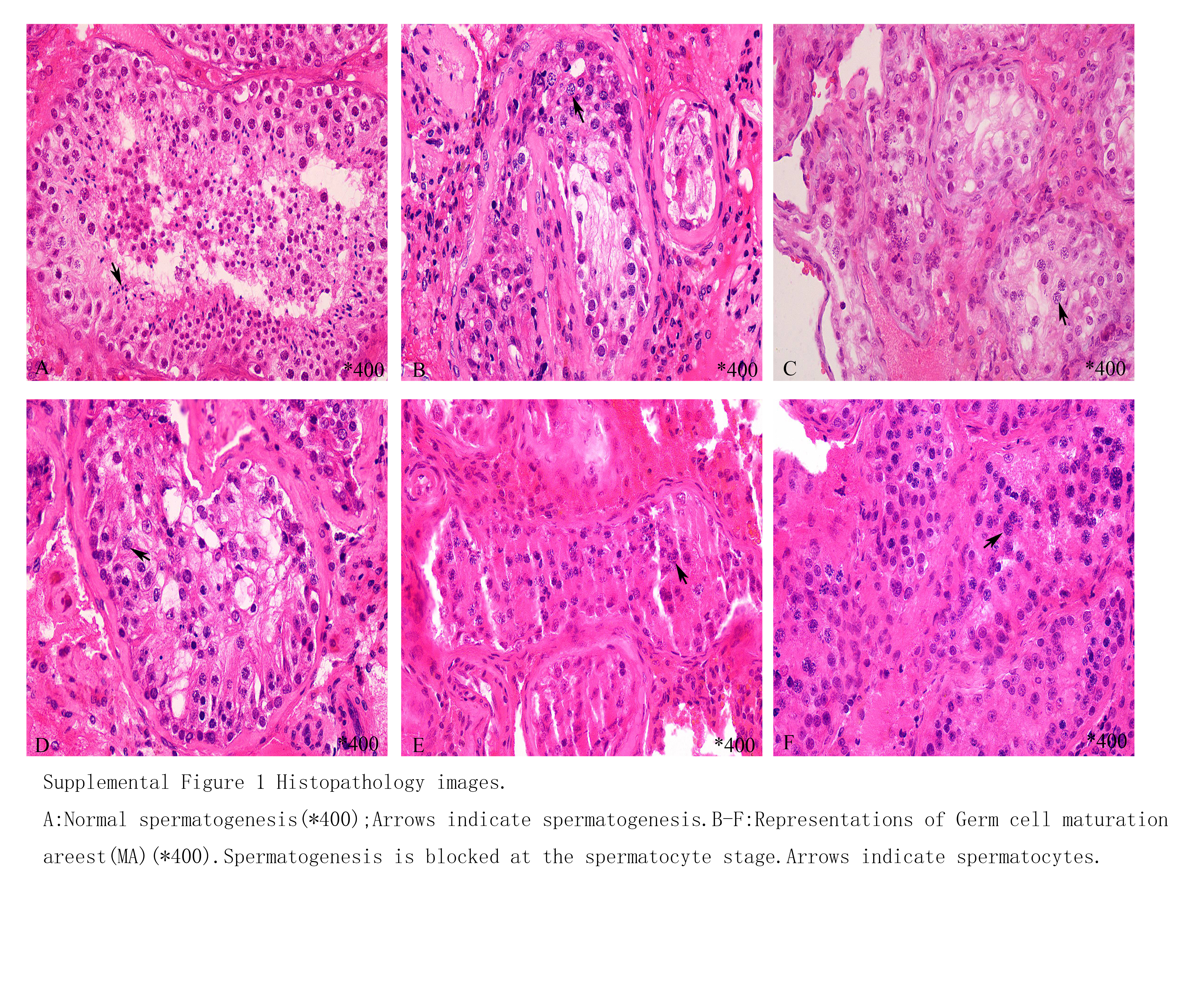

Supplement: Supplementary file 1 — Supplementary Information. [file 41598_2021_89559_MOESM1_ESM.jpg]
